# Hypnotism

**Published:** 1902-05-31

**Authors:** 


### HYPNOTISM.

In a lecture delivered before the King's College Medical Society Dr. Milne Bramwell[1] gave an interesting sketch of the subject of hypnotism. Describing the experiments of Forel, who till recently was medical director of the Burghölzi Asylum and one of the professors of the University of Zurich, he said that he succeeded in hypnotising nearly all his asylum attendants, both male and female, a large proportion of them becoming profound somnambules. For ten years experiments were made in regard to the use of hypnotism in the night watching of dangerous lunatics. Warders were hypnotised and trained to sleep by the bedside of these patients and to awake the instant they heard them attempt to get out of bed, the hypnotic suggestion being made use of to inhibit all sounds which had no reference to the duty laid upon them, and it was found that warders so hypnotised could perform night duty for six months and work hard all day without showing signs of fatigue. The results of these experiments were, it is said, uniformly successful, and no accident of any kind occurred. Forel was also able to excite and suppress menstruation by hypnotic and post-hypnotic suggestion. In regard to this and other applications of hypnotism, Dr. Bramwell refers to the method of Wetterstrand who, instead of restricting himself to suggestions made in the course of a short hypnotic trance, advocated the use of the curative effects of prolonged hypnotic sleep. Wetterstrand treated epilepsy and grave forms of nervous disorder by keeping the patients in the hypnotic trance for three or four weeks. Without rousing them the patients were fed at stated intervals, and the actions of the bowels and bladder were regulated by suggestion, and thus mental as well as physical rest was given, in addition to such therapeutic advantages as might be gained by aid of suggestion. Dr. Bramwell relates a case of his own in which a patient who had previously been cured of spinal neuralgia, insomnia, and dysmenorrhœa, suffered also from menorrhagia. The periods lasted a week and were excessive all the time, necessitating complete rest in bed. Suggestions given in the ordinary way having failed to relieve this, the patient was placed in a nursing home and kept in the hypnotic trance for three days before and five days during a period. "Since then menstruation has been normal in quantity and duration, and, instead of being confined to bed, the patient has been able to bicycle and take other active exercise." Dr. Milne Bramwell says that, although everyone cannot be deeply hypnotised, profound states are not necessary for the successful employment of suggestion, and the number of persons insusceptible to some degree of hypnotic influence is extremely small. Among other diseases he gives the following as those in which hypnotism has given good results: "Hysteria, neurasthenia, dipsomania and other drug habits, obsessions, moral perversities, and nervous tricks in children." We may add that, as far as the treatment of dipsomania is concerned, it seems not entirely impossible that some of the startling results which are said to have been obtained at certain institutions for the treatment of this condition, may have been the outcome of an unacknowledged but none the less effectual application of hypnotic suggestion.

[1] Clinical Journal, May 7 and 14.

